# OCF can repress tumor metastasis by inhibiting epithelial–mesenchymal transition involved in PTEN/PI3K/AKT pathway in lung cancer cells

**DOI:** 10.1371/journal.pone.0174021

**Published:** 2017-03-16

**Authors:** Ye Yang, Shuang Qiu, Lei Qian, Yuan Tian, Yingna Chen, Lei Bi, Weiping Chen

**Affiliations:** Department of Preclinical Medicine, Nanjing University of Chinese Medicine, Nanjing, Jiangsu, China; University of South Alabama Mitchell Cancer Institute, UNITED STATES

## Abstract

A component formula with definite compositions provides a new approach to treat various diseases. *Salvia miltiorrhiza* and *Panax ginseng* are widely used in China because of their antitumor properties. In the previous study, the optimizing component formula (OCF), prepared with salvianolic acids, ginsenosides, and ginseng polysaccharides (5, 10, and 5 mg·L^−1^, respectively) extracted from *S*. *miltiorrhiza* and *P*. *ginseng* on the basis of IC_50_ in lung cancer A549 cells and damage minimization on human bronchial epithelial cells in vitro. Currently, we also have demonstrated the inhibitory effect of OCF on A549 cell migration and invasion in vitro. According to Lewis lung cancer cells (LLC) allograft in C57BL/6 mice and A549 xenograft in nude mice experiment, we found that the anti-tumor and anti-metastasis effects of OCF treatment were related to the inhibition of epithelial–mesenchymal transition (EMT). Further studies showed that the inhibitory effect of OCF on EMT was associated with the PTEN/PI3K/AKT pathway. Therefore, all studies revealed that OCF could prevent cancer progression and tumor metastasis by inhibiting EMT involved PTEN/PI3K/AKT signaling pathway in lung cancer cells.

## Introduction

Lung cancer is a common disease and the leading cause of cancer-related deaths worldwide [1]. Non-small-cell lung cancer accounts for nearly 85% of all lung cancer cases reported [2]. In China, traditional Chinese medicine (TCM) has been applied to prevent and treat complex diseases through complicated synergistic or integrated actions. Experimental studies have revealed that component formulas with definite compositions can significantly inhibit lung cancer.

*Salvia miltiorrhiza* is an important traditional Chinese medicinal herb. According to TCM theory, *S*. *miltiorrhiza* promotes blood circulation and alleviates blood stasis. Therefore, this herbal medicine is widely utilized to prevent and treat various cardiovascular diseases, such as menstrual disorders and blood circulation disturbances. *S*. *miltiorrhiza* is also applied to treat hepatitis and hyperlipidemia, eliminate inflammation and inhibit tumor growth [3–4]. *S*. *miltiorrhiza* components can be classified as water-soluble (hydrophilic) phenolic compounds, such as rosmarinic acid, salvianolic acid A and B, lithospermic acid, and lipid-soluble (nonpolar) diterpenoidal known as tanshinones [5]. Phytochemical and pharmacological investigations have revealed that salvianolic acids and tanshinones are mainly responsible for the bioactive effects of *S*. *miltiorrhiza* [6]. Salvianolic acids are the main water-soluble ingredients of *S*. *miltiorrhiza*, they are widely utilized because of their cardiovascular, neural, and hepatic protection and antioxidant, anticoagulant, and cancer treatment effects [7]. Tanshinones are lipid-soluble constituents of *S*. *miltiorrhiza* and are characterized by potent antioxidant, anti-inflammatory, and anticancer activities in vitro and in vivo [8].

*Panax ginseng* is well-known traditional Chinese medicinal herb used to improve physical functions, possesses anti-cancer and anti-inflammatory effects, promote host resistance against infectious agents, affect metabolism, and enhance cellular glucose uptake [9–10]. *P*. *ginseng* contains many active components, such as ginsenosides, polysaccharides, essential oil, peptides, organic acids, vitamins, and minerals. Among these components, ginsenosides and polysaccharides have been reported as the major bioactive ingredients of ginseng in current functional investigations and clinical usage. Ginsenosides produce anticancer and immunomodulatory effects, whereas polysaccharides provide anticancer, radioprotective, anti-inflammatory, macrophage-stimulating, and immunomodulatory effects [11].

Although the anticancer effects of *S*. *miltiorrhiza* and *P*. *ginseng* have been reported, the combination of effective components to resist tumor has been rarely reported. In the previous study, an optimizing component formula (OCF) was prepared with 5 mg·L^−1^ salvianolic acids, 10 mg·L^−1^ ginsenosides, and 5 mg·L^−1^ ginseng polysaccharides from *S*. *miltiorrhiza* and *P*. *ginseng* according to the IC_50_ in lung cancer A549 cells and the damage minimization on normal BEAS-2B cells in vitro, which provides the basis for the exploration of antitumor effects in lung cancer [12]. In the present study, the inhibitory effect of OCF on cell invasion and migration in vitro and the progression of cancer and tumor metastasis in vivo were further elucidated. Considering the important role of the PTEN/PI3K/AKT signaling pathway in EMT and lung cancer, we analyzed the molecular mechanisms by which OCF elicits regulatory effects on EMT and tumorigenicity involved in the PTEN/PI3K/AKT pathway. This study revealed the possible anticancer mechanism of OCF on lung cancer.

## Materials and methods

### Materials

Salvianolic acids, tanshinones, ginsenosides, and ginseng polysaccharides were purchased from ZeLang Company (Nanjing, China). Standard ginsenosides Rg1, Re, Rd, and salvianolic acid B were obtained from National Institutes for Food and Drug Control (Beijing, China). Rosmarinic acid was procured from Nanjing Spring & Autumn Biological Engineering Company (Nanjing, China).

### Cell lines and culture

A549 (human non-small-cell lung cancer cell line) and mouse Lewis lung cancer cells (LLC) were purchased from Chinese Academy of Sciences, Shanghai Institute for Biological Sciences Cell Resource Center. A549 cells were cultured in Dulbecco’s modified Eagle’s medium (DMEM)/F12 (Hyclone, Thermo Fisher Scientific Inc., Massachusetts, USA), whereas BEAS-2B and LLC cells were cultured in DMEM (Hyclone, Thermo Fisher Scientific Inc., Massachusetts, USA) containing 10% fetal bovine serum (FBS; Gibco Life Technologies, Grand Island, USA), 100 unit/mL penicillin, and 100 μg/mL streptomycin (Hyclone, Thermo Fisher Scientific Inc., Massachusetts, USA). Cultures were maintained at 37°C in a humidified atmosphere of 95% air and 5% CO_2_.

### Animals and ethics statement

Four-week-old female C57BL/6 mice and BALB/c athymic nude mice were purchased from Beijing Vital River Laboratory Animal Technology Co., Ltd. (Beijing, China) and Nanjing Biomedical Research Institute of Nanjing University (Nanjing, China), respectively. All of the mice were maintained in specific pathogen-free (SPF) conditions with uniform environment (12 h light-dark cycle with constant room temperature) and fed with standard rodent chow and water. All procedures involving animals were approved by the Institutional Animal Care and Use Committee at Nanjing University of Chinese Medicine and were carried out in accordance with the Guide for the Care and Use of Laboratory Animals.

### Content determination of total salvianolic acids, ginsenosides, and ginseng polysaccharides

The total salvianolic acid was measured through NaNO_2_-Al(NO_3_)_3_ colorimetric method. In brief, the proper concentration sample in a 15 mL centrifuge tube was mixed with 2.5 mL of 1% NaNO_2_ and placed in the dark for 10 min. Afterward, 0.25 mL of 20% Al(NO_3_)_3_ was added to the mixture and the tube was placed in the dark for 10 min. The sample was precisely mixed with 4 mL of 1 mol·L^−1^ NaOH and placed in the dark for 15 min. Absorbance was determined at 493 nm. Salvianolic acid B reference solutions (0.4 g·L^−1^) were precisely weighed as 0, 0.07, 0.21, 0.35, 0.49, 0.63, and 0.77 mL to prepare a calibration curve.

The content of ginsenosides was determined by vanillin-sulfuric acid colorimetric method. In brief, the proper concentration sample in a 10 mL centrifuge tube was mixed with 0.5 mL of 1% vanillin perchloric acid solution and placed at 60°C for 10 min. After the sample was placed in cool water for 2 min, 5 mL of 77% H_2_SO_4_ was precisely added. Absorbance was determined at 540 nm. Ginsenoside Re reference solutions (1 g·L^−1^) were precisely weighed as 0, 0.04, 0.06, 0.08, 0.1, 0.12, 0.14, and 0.16 mL to prepare a calibration curve.

The content of ginseng polysaccharides was measured by phenol-sulfuric acid colorimetric method. In brief, the proper concentration sample in a 10 mL centrifuge tube was mixed with 1.0 mL of 5% phenol solution and placed at 40°C for 30 min. After the sample was placed in cool water for 10 min, absorbance was determined at 490 nm. Glucose reference solutions 0.5 mL (0.01, 0.02, 0.04, 0.06, 0.08, and 0.1 g·L^−1^) were weighed to prepare a calibration curve.

### HPLC analysis

Salvianolic acids and ginsenosides were identified by comparing the retention time of the samples with that of standard samples by using a UPLC system (Agilent 1290 Infinity UPLC, Agilent Technologies, Santa Clara, USA). A Kromasil C_18_ column (4.6 mm × 250 mm, 5 μm) was used to detect salvianolic acids. Separation was achieved using a gradient elution with methanol (mobile phase A) and 0.1% formic acid aqueous solution (mobile phase B): 0–40.0 min and 37.0%–45.0% (v/v) solvent A. The flow rate was 1.0 mL·min^−1^. The column temperature was 30°C. All of the samples were filtered through a membrane filter (0.22 μm). The injection volume was 10 μl. The UV detection wavelength was 286 nm.

A ZORBAX SB C_18_ column (2.1 mm × 50 mm, 1.8 μm) was used to detect ginsenosides. Separation was achieved by using a gradient elution with acetonitrile (mobile phase A) and water (mobile phase B): 0–17.0 min, 17% solvent A; 17.0–19.0 min, 17%–32.0% (v/v) solvent A; 19.0–29.0 min, 32.0% solvent A; and 29.0–30.0 min, 32.0%–17.0% (v/v) solvent A. The flow rate was 0.4 mL·min^−1^. The column temperature was 30°C. All of the samples were filtered through a membrane filter (0.22 μm). The injection volume was 10 μl. The UV detection wavelength was 203 nm.

### Wound healing scratch assay

A549 cells were seeded in 6-well plates with a culture medium containing 1 × 10^5^ cells in each well and accomplished in triplicate in the wound healing scratch assay. After 24 h of incubation, the confluent cell layers were scratched in line by using a sterile pipette tip of 200 μL. The cells were gently rinsed twice with PBS and cultivated with the OCF and in the culture medium without FBS. The same area of the gap was imaged at 0, 24, and 48 h and quantified using Image J software. The distance between initial and final areas was used to determine the migratory ability of A549 cells.

### High-Content Screening (HCS) analysis of cellular motility

We used a cell motility kit (Thermo Fisher Scientific Inc., Massachusetts, USA) to detect the effect of cell motility and to confirm the result of the wound healing scratch assay in accordance with the manufacturer’s instructions with some modifications. The cells used to detect migration were plated on the lawn of microscopic fluorescent microspheres. The cells pushed aside the microspheres as they moved across to clear the tracks behind them. The blue fluorescent microspheres formed a background, and the cells were stained and identified with rhodamine-conjugated phalloidin. The track area is proportional to the magnitude of cell movement [13–14]. In brief, the blue fluorescent microspheres were added to a 96-well plate coated with collagen I. A549 cells were brought into suspension and seeded in the treated 96-well plate. Each well containing 5 × 10^2^ cells was incubated at 37°C in a humidified atmosphere of 95% air and 5% CO_2_ for 24 h. The cells were then exposed to the OCF and the culture medium without serum for 48 h. Each group was set up for three repetitions. The cells were treated with 5.5% warmed fixation solution and permeability buffer and stained with rhodamine-conjugated phalloidin. Afterward, the plates were placed in an AssayScan HCS reader (Thermo Fisher Scientific Inc., Massachusetts, USA) to acquire the images and then analyzed with Cell Motility Bioapplication.

### Real-time monitoring of cellular invasion

Cell invasion was performed with a real-time cell analyzer (RTCA) by using cell invasion and migration (CIM) plates. This instrument utilizes electrical impedance to non-invasively quantify adherent cell proliferation in real time [15]. The system measures electrical impedance across microelectrodes integrated into the bottom of tissue culture CIM plate. Cell-sensor impedance is expressed in an arbitrary unit called cell index (CI). CI provides quantitative information on the biological status of cells, including cell number, viability, and morphology. The RTCA software supplied by the manufacturer was used to analyze these measurements and calculate the doubling time of the cells on the basis of CI [16–17]. In brief, a layer of Matrigel gel was added to the upper chamber of the CIM plates, and the A549 cells were seeded on the upper chamber of the plates in the culture medium with each well containing 2 × 10^4^ cells. The serum-containing medium was filled in the bottom chambers of the CIM plates to promote migration across membranes and toward the serum gradient. CIM plates were then transferred to the RTCA instrument for automated real-time monitoring at 37°C in a humidified atmosphere of 95% air and 5% CO_2_. The cells were incubated for 24 h and then exposed to the OCF and culture medium without serum for another 72 h. Each group was prepared in triplicate. The cell invasion curves were automatically recorded on xCELLigence System in real time.

### qRT-PCR

Total RNA was extracted from homogenized cancer tissues by using Trizol reagent (Takara, Shiga, Japan), and 1 μg of RNA was used as a template for reverse transcription (Vazyme Biotech, Nanjing, China) in accordance with the manufacturer’s instructions. SYBR^®^ Green Master Mix (High ROX Premixed; Vazyme Biotech, Nanjing, China) was used for amplification in Real-time QPCR System (Applied Biosystems, Foster City, CA, USA). The primer sequences were as follows: E-cadherin, forward primer 5′-AAGCCTCAGGTCATAAACATCATTG-3′ and reverse primer 5′-TTCTTGGGTTGGGTCGTTGTAC-3′; N-cadherin, forward primer 5′-TGGCGGAGATCCTACTGGACG-3′ and reverse primer 5′-TGACTGAGGCGGGTGCTGAA-3′; fibronectin, forward primer 5′-GGGAGAAGTATGTGCATGGTGTC-3′ and reverse primer 5′-TTGGAAATGTGAGATGGCTGTG-3′; and β-actin, forward primer 5′-CCCATGCCATCCTCCGTCTG-3′ and reverse primer 5′-TCTCGGCTGTGGTGGTGAAG-3′. The results were analyzed with the 2^-ΔΔCT^ method by using β-actin as an internal normalization control. qRT-PCR was repeated thrice for each sample. All primers were purchased from Genscript (Nanjing, China).

### Immunohistochemical staining analysis

Immunohistochemistrical assay was performed in tumor tissues in accordance with a standard immunostaining protocol. Paraffin sections were deparaffinized in xylene, washed in decreasing alcohol concentrations, and rehydrated in deionized water. Antigen retrieval was performed in 10 mmol·L^-1^ citrate buffer for 2 min at 100°C. Endogenous peroxidase activity was blocked by immersing the slides in 3% hydrogen peroxide for 10 min. The slides were incubated at 4°C overnight with different rabbit polyclonal antibodies at assay dependent concentration. Primary antibodies used include E-cadherin antibody (1:50) (Beijing ZhongShan Biotechnology Co. Ltd., Beijing, China); N-cadherin (1:200), Fibronectin (1:100), PTEN antibody (1:100) (Abcam, MA, USA); and phosphorylated AKT (p-AKT, Ser 473) antibody (1:100) (Cell Signaling Technology, Beverly, MA). The slides were then incubated with polyperoxidase-anti-mouse/rabbit IgG (Beijing ZhongShan Biotechnology, Beijing, China). Peroxidase reaction was performed using 3,3′-diaminobenzidine (DAB; Beijing ComWin Biotech, Beijing, China). The slides were visualized with 3,3′-diaminobenzidine (DAB), counterstained with hematoxylin, mounted with neutral gum (Beijing ComWin Biotech, Beijing, China), and photographed using a microscope equipped with a camera (Zeiss, Germany). The slides were reviewed and quantified in a double-blind manner by two investigators using Image Pro Plus.

### Western blot analysis

Total protein was extracted from the treated cells or homogenized cancer tissues with RIPA cell lysis buffer supplemented with phenylmethylsulfonyl fluoride (PMSF), proteinase, and phosphatase inhibitors. Protein concentrations were quantified using a BCA protein assay kit in accordance with the manufacturer’s instructions. Equal amounts of protein were separated by 10% sodium dodecyl sulfate-polyacrylamide gel electrophoresis and transferred to polyvinylidene fluoride (PVDF) membranes (Millipore, Bedford, MA, USA). The membranes were blocked with TBST containing 3% bovine serum albumin (BSA) at room temperature for 1 h. The membranes were then incubated overnight at 4°C with appropriately diluted primary antibodies, E-cadherin antibody (1:1000), N-cadherin (1:1000), fibronectin (1:1000), PTEN (1:1000), beta actin antibody (1:3000), phosphorylated AKT (1:2000), and AKT antibody (1:1000) (Cell Signaling Technology, Beverly, MA). The membranes were extensively washed with TBST buffer (Tris Buffer Saline containing Tween-20) and incubated for 1 h with goat anti-rabbit IgG conjugated to horseradish peroxidase (Beyotime Institute of Biotechnology, Shanghai, China). Target protein bands were visualized with enhanced chemiluminescence ECL reagents (Beijing ComWin Biotech, Beijing, China).

### In vivo tumor allograft and xenograft experiment

The LLC and A549 cells were collected for tumor allograft and xenograft experiments. All animal studies were conducted to induce tumors. In the tumor allograft experiment, approximately 3 × 10^6^ LLC cells in 0.2 mL normal saline (NS) were injected subcutaneously into the right lateral axilla of normal C57BL/6 mice. One day after LLC cell inoculation, the mice were randomly divided into four groups (10 mice per group). For the following days, the NS-injected mice were used as the control group, whereas the drug groups were administered with different doses of OCF via intraperitoneal injection once a day for 26 consecutive days. The high-dose OCF group was treated with 80 mg/kg salvianolic acids, 160 mg/kg ginsenosides, and 80 mg/kg ginseng polysaccharides. The middle-dose OCF group was administered with 40 mg/kg salvianolic acids, 80 mg/kg ginsenosides, and 40 mg/kg ginseng polysaccharides. The low-dose OCF group received 20 mg/kg salvianolic acids, 40 mg/kg ginsenosides, and 20 mg/kg ginseng polysaccharides.

In the tumor xenograft experiment, 3 × 10^6^ A549 cells were subcutaneously injected into the right lateral axilla of the nude mice. The mice were randomly divided into four groups (8 mice per group) when the mean volumes of tumors were between 50 and 100 mm^3^. For the following days, the NS-injected mice were used as the control group, whereas the drug groups were administered with different doses of OCF via intraperitoneal injection once a day for 18 consecutive days.

The weight and diameters of developing tumors were evaluated every 3 days. Tumor volumes were calculated according to the following equation: tumor volume (mm^3^) = *π* × (length × width^2^) /6, where length and width were measured as the maximum and the minimum tumor diameter, respectively [18]. At the end of the experiment, the animals were sacrificed with cervical dislocation, and their lungs and tumors were harvested. The lungs were used to count the lung metastatic foci and analyzed by H&E staining. The tumors were subjected to IHC, qRT-PCR, and western blot tests.

### Statistical analysis

Data were analyzed using SPSS 13.0 (SPSS, Chicago, IL, USA). Each experiment was performed in triplicate. Data were expressed as mean ± SD. One-way ANOVA was performed to compare between groups, and least significant difference test was conducted to compare between experimental groups. *P* < 0.05 was considered statistically significant. Mean values were used for drawing the graphs.

## Results

### Quantification of salvianolic acids, ginsenosides, and ginseng polysaccharides

Standard curves were drawn by using the content of reference substance (μg) as the X-axis and absorbance A as the Y-axis. The linear regression equation and related coefficient of salvianolic acids, ginsenosides, and ginseng polysaccharides are shown in Table 1. The contents of total salvianolic acids, ginsenosides, and ginseng polysaccharides were 86.66%, 83.81%, and 72.72%, respectively.

**Table 1:** Regression equation of salvianolic acids, ginsenosides, and ginseng polysaccharides.

Active components	Regression equation	r^2^	Linear range (μg)
Salvianolic acids	Y = 0.004X + 0.025	0.999	28.14–309.54
Ginsenosides	Y = 0.004X − 0.049	0.999	40–160
Ginseng polysaccharides	Y = 0.01X + 0.044	0.999	5–50

**Fig 1:**
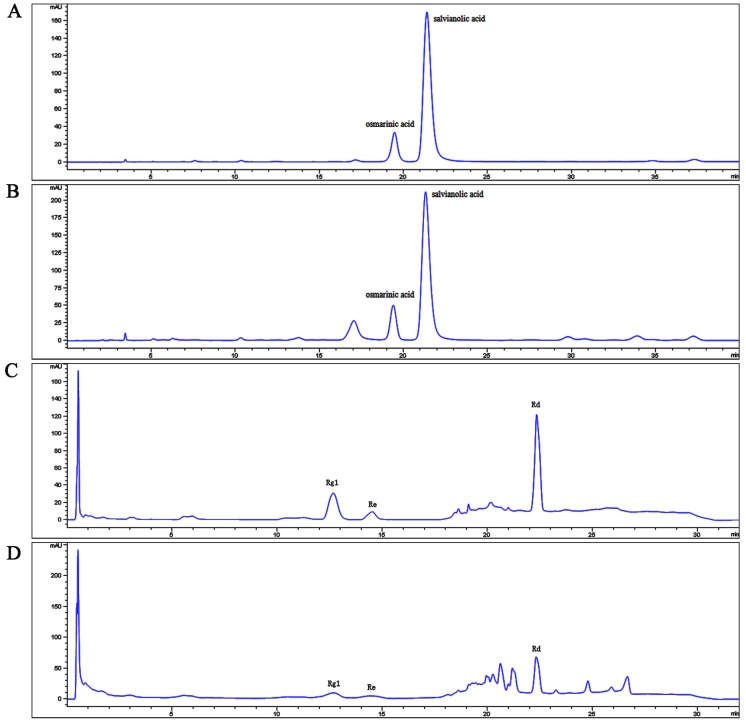
Quantification of salvianolic acids and ginsenosides. (A) HPLC chromatogram of reference compounds in salvianolic acids. (B) HPLC chromatogram of salvianolic acids. (C) HPLC chromatogram of reference compounds in ginsenosides. (D) HPLC chromatogram of ginsenosides.

The HPLC chromatographic profiles of salvianolic acids and ginsenosides are shown in Fig 1A and 1D. Salvianolic acid B and rosmarinic acid were the components of salvianolic acids with the concentrations of 68.01% and 12.24% as the positive controls according to China Pharmacopoeia 2015 Edition (salvianolic acid B should not be less than 5.0% and rosmarinic acid should not be less than 0.50%). The total amount of Rg1, Re, and Rd in ginsenosides was 20.83% as the positive controls according to China Pharmacopoeia 2015 Edition (the total amount of Rg1, Re, and Rd in ginsenosides was 15%–25%).

### OCF inhibited the migration and invasion of A549 cells in vitro

**Fig 2:**
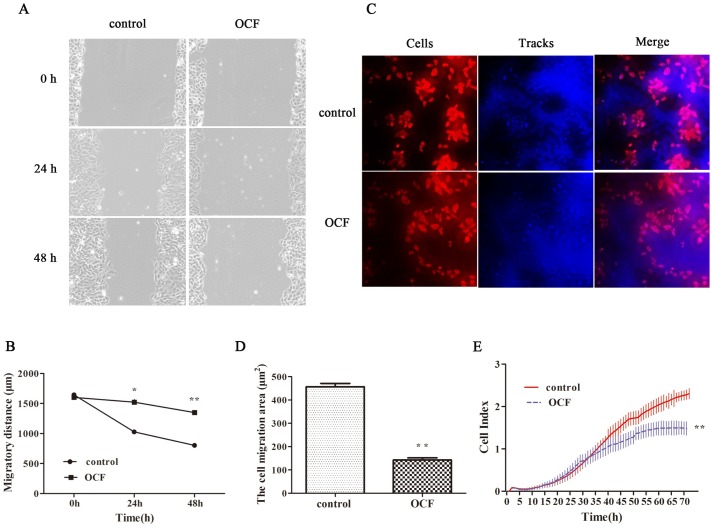
OCF inhibited the migration and invasion of A549 cells in vitro. (A) Representative images of the same wounded area with different treatments at 0, 24, and 48 h after scratching (100×). (B) The migratory distance of scratch with different treatments at 0, 24, and 48 h after scratching. Migratory distance was measured and quantified by Image J. (C) Representative images of cell migration in A549 cells (200×). Cells were stained with rhodamine-conjugated phalloidin and characterized by red fluorescence. The blue fluorescent microspheres formed a background. The cells pushed aside the microspheres as they moved across to clear the tracks behind them. Tracks are generated by cells and represent the movement of cells. (D) The tracks generated by cells were quantified by the Cell Motility Bioapplication. (E) RTCA dynamic monitoring invasion of A549 cells treated with OCF. A549 cells were seeded and cultured for 24 h on CIM plates with the culture medium. The cells were then treated with OCF for another 72 h. Cell growth curves were automatically recorded on the xCELLigence System in real time. Results were expressed as mean ± SD, **P<0.01 and *P<0.05 compared with the control group (n = 3).

Wound healing scratch assay was performed to investigate the effects of OCF on the migration of A549 cells. In Fig 2A and 2B, the migratory distance of OCF at 48h was significantly broader than that of the control group (*P*<0.01). Cell migration was also performed by using a cell motility kit and detected through HCS. The cells pushed aside the microscopic fluorescent microspheres as they moved across the lawn to clear the tracks behind them. The number and track areas of the migrating cells were calculated with HCS Cell Motility BioApplication to assess cell migration. In Fig 2C and 2D, the track area of the A549 cells treated with the OCF at 48h was significantly smaller than that of the control group (*P*<0.01). We used the RTCA system with CIM plates to verify the effect of the OCF on cell invasion (Fig 2E). The cells that can migrate from the upper chamber through the membrane and into the bottom chamber come in contact and adhere to the sensors; as a result, impedance and Cell Index read-outs were increased [19]. Compared with the control group, the OCF remarkably reduced the number of cells passing through the membrane (*P*<0.01). These results indicated that OCF could suppress the migration and invasion of A549 cells.

### OCF inhibited the tumor growth and metastasis of LLC-allograft mice

**Fig 3:**
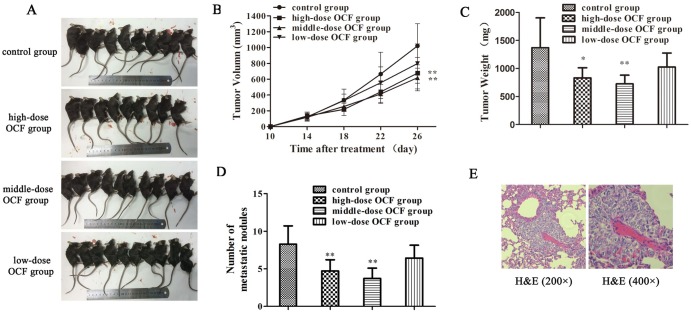
OCF inhibited the tumor growth and metastasis of LLC-allograft mice in vivo. (A) Mice were injected subcutaneously with LLC cells. At the end of the experiment, the mice were sacrificed and photographed. (B) Tumor growth curve of LLC-allograft mice treated with NS and different doses of OCF. (C) Tumor weight of LLC-allograft mice treated with NS and different doses of OCF. (D) Lung metastatic nodules of LLC-allograft mice treated with NS and different doses of OCF. (E) H&E staining of lung tissues from the control group of LLC-allograft mice (200× and 400×). The high-dose OCF group was treated with 80 mg/kg salvianolic acids, 160 mg/kg ginsenosides, and 80 mg/kg ginseng polysaccharides; the middle-dose OCF group was administered with 40 mg/kg salvianolic acids, 80 mg/kg ginsenosides, and 40 mg/kg ginseng polysaccharides; the low-dose OCF group received 20 mg/kg salvianolic acids, 40 mg/kg ginsenosides, and 20 mg/kg ginseng polysaccharides. Data were expressed as mean ± SD, n = 10. **P < 0.01 and *P < 0.05 compared with the control group.

The encouraging results from the in vitro studies indicated that OCF could effectively inhibit A549 cell migration and invasion. We further investigated the effect of OCF in vivo. The LLC cells were injected subcutaneously into the right lateral axilla of normal C57BL/6 mice and treated with NS and different doses of OCF for 26 consecutive days to investigate the anti-metastatic effect of OCF on LLC cells in vivo. In Fig 3A and 3B, tumor volume was 1020.29 ± 260.75 mm^3^ in the control group, 675.77 ± 183.57 mm^3^ in the high-dose OCF group, 617.17 ± 141.93 mm^3^ in the middle-dose OCF group, and 797.41 ± 214.35 mm^3^ in the low-dose OCF group. The average volume of tumors in middle dose OCF group was significantly lower (*P* < 0.01) than the average volume of tumors in the control group. OCF significantly reduced the tumor weight relative to that of the control group (Fig 3C). The inhibition rates of high-, middle-, and low-dose OCF groups were 33.77%, 39.51%, and 21.84%, respectively. The number of metastatic nodules was significantly decreased in high-, middle- dose OCF group compared with that of the control group (*P*<0.01) (Fig 3D). Fig 3E showed H&E staining of lung tissues from the control group of LLC-allograft mice. The results of LLC tumor allograft experiment confirmed that OCF could suppress the tumor growth and metastasis of LLC-allograft mice in vivo.

### OCF suppressed tumor growth on A549 tumor xenograft

**Fig 4:**
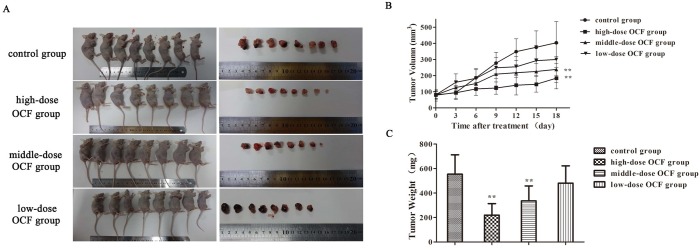
OCF inhibited the growth of A549 tumor xenograft in vivo. (A) Mice were injected subcutaneously with A549 cells. At the end of the experiment, the mice were sacrificed and photographed; tumors were isolated from the mice. (B) Tumor growth curve of A549-xenograft mice treated with NS and different doses of OCF. (C) Tumor weight of A549-xenograft mice treated with NS and different doses of OCF. The high-dose OCF group was composed of 80 mg/kg salvianolic acids, 160 mg/kg ginsenosides, and 80 mg/kg ginseng polysaccharides; the middle-dose OCF group was composed of 40 mg/kg salvianolic acids, 80 mg/kg ginsenosides, and 40 mg/kg ginseng polysaccharides; the low-dose OCF group was composed of 20 mg/kg salvianolic acids, 40 mg/kg ginsenosides, and 20 mg/kg ginseng polysaccharides. Data were expressed as mean ± SD, n = 8. **P < 0.01, *P < 0.05 compared with the control group.

A549-xenograft mouse model was established to investigate the antitumor activity caused by OCF treatment in vivo. A549 cells were injected subcutaneously into the right lateral axilla of the nude mice. The mice were treated with NS and different OCF doses when the tumor volume reached 50–100 mm^3^ for 18 consecutive days. In Fig 4A and 4B, tumor volumes were 403.65 ± 130.38 mm^3^ in the control group, 183.90 ± 65.21 mm^3^ in the high-dose OCF group, 238.39 ± 80.02 mm^3^ in the middle-dose OCF group, and 299.85 ± 98.00 mm^3^ in the low-dose OCF group. The tumor growth curve indicated that OCF significantly reduced the tumor volume in a dose-dependent manner compared with that of the control group. The average volume of tumors in the high-dose OCF group increased at a significantly slower (P < 0.01) rate than the average volume of tumors in the control group. OCF significantly decreased the tumor weight in a dose-dependent manner compared with that of the control group (Fig 4C). The average weight of tumors in the high-dose OCF group increased at a significantly slower rate than the average weight of tumors in the control group (P < 0.01). The inhibition rates of high-, middle-, and low-dose OCF groups were 54.44%, 40.94%, and 25.71%, respectively. These results demonstrated that OCF could inhibit the tumor growth of A549-xenograft mice in vivo.

### OCF regulated EMT-associated marker expression

**Fig 5:**
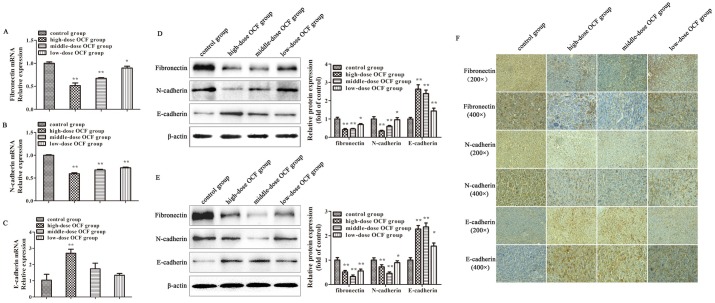
OCF regulated the expression of EMT-associated genes. mRNA levels of (A) fibronectin, (B) N-cadherin, and (C) E-cadherin measured by qRT-PCR from the tumor tissues of A549-xenograft mice. (D) Protein levels of fibronectin, N-cadherin, and E-cadherin were determined through Western blot assay from the tumor tissues of A549-xenograft mice. (E) Protein levels of fibronectin, N-cadherin, and E-cadherin were measured by Western blot assay from the tumor tissues of LLC-allograft mice. (F) Immunohistochemistry for fibronectin, N-cadherin, and E-cadherin protein expression in the tumor tissues of LLC-allograft mice (200× and 400×). Data were expressed as mean ± SD of three experiments. **P < 0.01, *P < 0.05 compared with the control group.

EMT plays a pivotal role in promoting tumor invasion and metastasis. To investigate whether OCF decreases cancer migration and invasion by inhibiting EMT, we detected the mRNA levels of several EMT-associated markers in the tumor tissues of A549-xenograft mice. In Fig 5A–5C, qRT-PCR results revealed that OCF significantly decreased the mRNA levels of fibronectin and N-cadherin (*P* < 0.01). By contrast, high-dose OCF group increased the mRNA level of the epithelial marker E-cadherin in tumor tissues of A549-xenograft mice (*P* < 0.01). We further investigated the changes in these EMT-associated markers protein levels through western blot and immunohistochemistry analysis. In Fig 5D and 5E, western blot analysis demonstrated that OCF increased the protein level of E-cadherin and decreased the protein levels of fibronectin and N-cadherin in the tumor tissues of A549-xenograft mice and LLC-allograft mice (*P* < 0.01). We also confirmed the equal results by IHC in the tumor tissues of LLC-allograft (Fig 5F).

### OCF inhibited the EMT involved in the PTEN/PI3K/AKT signaling pathway

**Fig 6:**
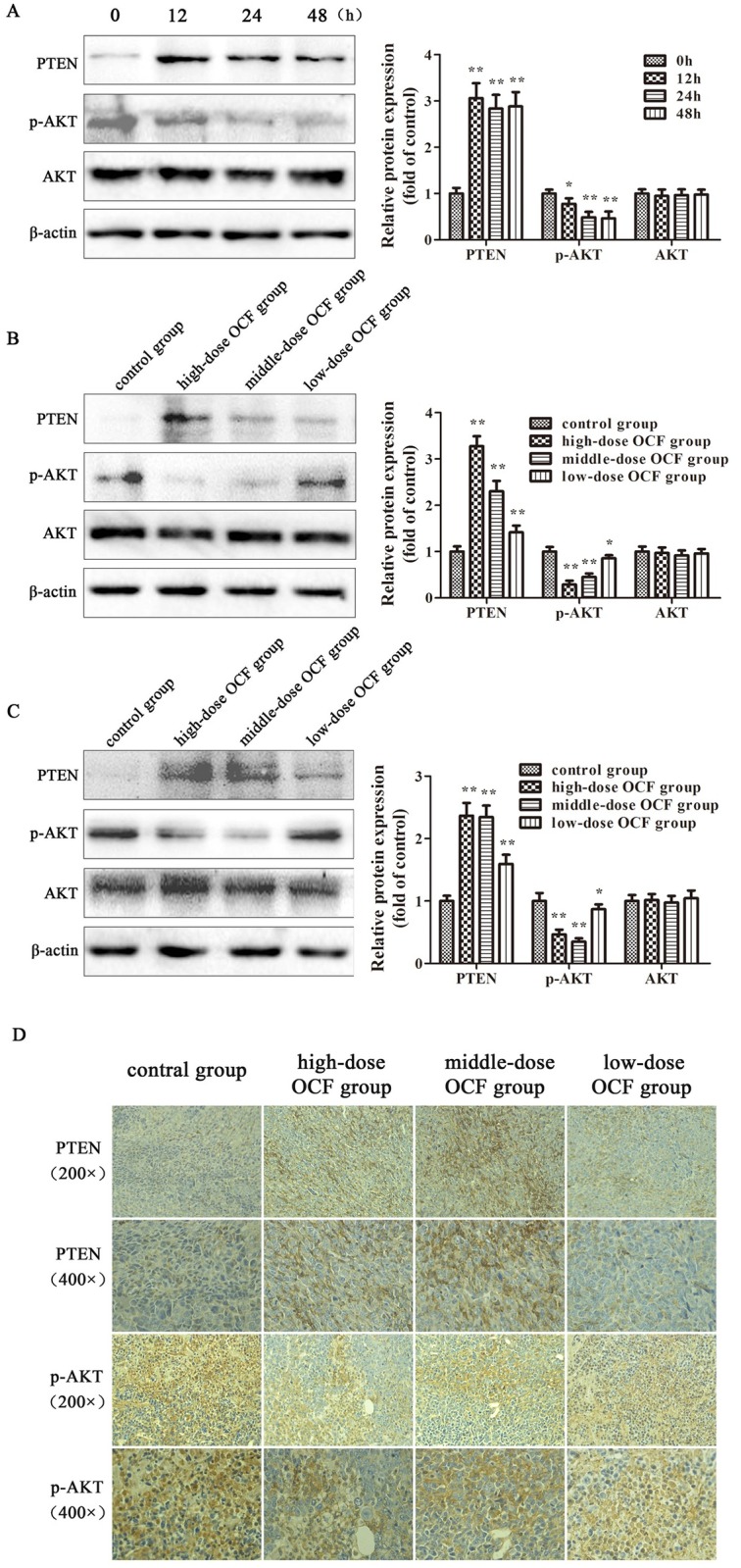
OCF regulated the protein expression of PTEN//PI3K/AKT signaling pathway. (A) Protein levels of PTEN, p-AKT, and AKT were measured from A549 cells treated with OCF for 12, 24, and 48 h. (B) Protein levels of PTEN, p-AKT, and AKT were measured by Western blot assay from the tumor tissues of A549-xenograft mice. (C) Protein levels of PTEN, p-AKT, and AKT were determined through Western blot assay from the tumor tissues of LLC-allograft mice. (D) Immunohistochemistry for PTEN and p-AKT protein expression in the tumor tissues of LLC-allograft mice (200× and 400×). Data were expressed as mean ± SD of three experiments. **P < 0.01, *P < 0.05 compared with the control group.

The PTEN/PI3K/AKT pathway was involved in the occurrence of EMT [20–21]. To determine whether the inhibitory effect of OCF on EMT is related to the PTEN/PI3K/AKT signaling pathway, we evaluated the effects of OCF on the expression of PTEN, p-AKT, and AKT by Western blot. In Fig 6A, OCF could increase the protein levels of PTEN in A549 cells after the OCF treatment was administered in 12, 24, and 48 h (*P* < 0.01). We further investigated the effect of OCF on the other important members of the PTEN pathway. We found that the protein level of p-AKT was downregulated after the same OCF treatment was administered (*P* < 0.01), whereas the protein level of AKT remained unchanged. We subsequently evaluated the effect of OCF on the levels of PTEN in the tumor tissues of A549-xenograft mice and LLC-allograft mice. In Fig 6B and 6C, OCF could increase the level of PTEN in tumor tissues (*P* < 0.01). The protein level of p-AKT was downregulated and the protein level of AKT remained unchanged. We further performed IHC to verify the expression of PTEN and p-AKT in the tumor tissues of LLC-allograft mice. In Fig 6D, OCF could increase the level of PTEN and decrease the level of p-AKT. These results suggested that OCF could inhibit EMT by increasing PTEN and inactivating PI3K/AKT signaling.

## Discussion

TCM is based on a sophisticated system of medical theory with complex components and multiple activities which enhances beneficial effects and reduced side effects on complex diseases [22–23]. The pharmacological effects of TCM should be investigated by using effective components as the fundamental elements of TCM. Effective components are isolated and purified chemical compounds from TCM with similar molecular structures, these compounds represent the chemical substances of TCM that effectively improve the efficacy and quality control while reduce side effects [24].

The active compounds of *S*. *miltiorrhiza* and *P*. *ginseng* may be used clinically for antitumor therapy. Previously, the proportions of several effective components, such as salvianolic acids, tanshinones, ginsenosides, and ginseng polysaccharides, of *S*. *miltiorrhiza* and *P*. *ginseng* were optimized by conducting orthogonal experiments to selectively inhibit A549 cells but minimize the damage to normal BEAS-2B cells. According to the results of orthogonal experiments, the dose distribution among salvianolic acids, ginsenosides, and ginseng polysaccharides were 5, 10, and 5 mg∙L^−1^, respectively. The optimizing component formula was named the OCF. Interestingly, the dose of tanshinones in OCF was zero. The results indicated that the toxicity of tanshinones in BEAS-2B cells might be higher than that in A549 cells.

In the present study, we found that the OCF inhibited the migration and invasion of A549 cells. Wound healing scratch assay and Cell Motility HitKit assay were performed to demonstrate the effect of OCF on A549 cell migration. Scratch experiment is a classic method to detect cell migration [25]. Cell Motility HitKit is designed to facilitate the quantification of cell migration. In our study, OCF could effectively reduce the A549 cell migration. To monitor cellular invasion responses, we used the RTCA xCELLigence system with CIM plates. On the basis of the results, we concluded that OCF effectively inhibited A549 cell invasion.

To verify the OCF in vitro findings of the inhibition of migration and invasion in A549 cells can occur in vivo, we investigated the effects of OCF in LLC-allograft in C57BL/6 mice and A549 xenograft in nude mice. The LLC cell line is a well-established mouse cancer model, which is extensively applied as a transplantable malignancy model in syngeneic C57BL/6 mice [26]. Human tumor xenograft model has been used extensively to predict the anti-tumor efficacy and reveal the pathogenesis of related diseases [27]. The body weight, hair coats, and other overall behavioral activities were similar among the groups before completion of the experiments in LLC allograft and A549 xenograft models. This finding indicated that OCF did not elicit major side effects on C57BL/6 and nude mice. Our current data showed that OCF significantly inhibit tumor growth and tumor metastasis. There are two different dose which were effected on the two animal models. High-dose of OCF group significantly inhibited the progression of tumor compared with A549-xenograft mice in control group, whereas in LLC-allograft mice, middle-dose OCF group exhibited the best result. This finding needs to be explored in the future.

We demonstrated OCF repress cell invasion and migration in A549 cells in vitro and repress cancer progression and tumor metastasis in vivo. Migration and invasion are closely related to EMT that plays a pivotal role in the metastatic progression of lung cancer [28–29]. Epithelial cells lose their characteristics during EMT and switch to mesenchymal phenotype; the new phenotype increases the migratory capacity and invasiveness of cells and thus facilitates tumor progression [30–31]. The molecular mechanism of EMT involves the downregulation of E-cadherin and upregulation of mesenchymal markers, such as N-cadherin, fibronectin, and vimentin, or signal transduction proteins, such as Snail and Twist [32]. The occurrence of EMT is associated with several complex signal transduction pathways, including the PTEN/PI3K/AKT signaling pathway [33]. We hypothesized that OCF could decrease cancer migration and invasion by inhibiting EMT. To verify this hypothesis, we detected the expression of E-cadherin, N-cadherin, and fibronectin in tumor tissues through PCR, Western blot, and immunohistochemistry. Our results demonstrated that OCF increased the expression of E-cadherin, which is often lost in metastatic tumors as a feature of EMT. By contrast, OCF decreased the expression of N-cadherin and fibronectin. Therefore, OCF can inhibit tumorigenesis and metastasis in vivo by suppressing EMT.

PTEN as a phosphatase for phosphoinositol lipids has been identified as a multifunctional tumor suppressor frequently implicated in many kinds of cancers [34–35]. The deletion or inactivation of PTEN results in tumorigenesis in a large number of cancers, including lung cancer [36]. PTEN dephosphorylates phosphatidylinositol-3,4,5-trisphosphate (PIP3) to generate phosphatidylinositol 4,5-bisphosphate (PIP2) and thus negatively regulates the PTEN/PI3K/AKT signaling pathway [37]. AKT, a serine/threonine protein kinase, is a critical downstream target of PI3K, which plays an important role in cell growth modulation, angiogenesis, migration, and metabolism [38]. The mechanism by which PTEN inhibits tumor cell growth and invasion is associated with the intervention of the oncogenic PTEN/PI3K/AKT signaling pathway [39]. To determine whether OCF can inhibit EMT through the PTEN/PI3K/AKT signaling pathway, we evaluated the effect of OCF on increasing the expression of PTEN and repressing p-AKT, as indicated by Western blot and immunohistochemistry analyses. These results suggested that the suppression of the PI3K/AKT signaling pathway was modulated by increasing PTEN expression in tumor tissues by OCF.

In summary, OCF inhibited the migration and invasion of A549 cells in vitro and reduced the tumor growth in LLC allograft in C57BL/6 mice and A549 xenograft in nude mice in vivo. OCF also suppressed lung cancer cell invasion and metastasis by inhibiting EMT in vitro and in vivo through the modulation of the PTEN/PI3K/AKT signaling pathway. The results allow for providing novel insights into the inhibitory mechanism of EMT and cancer metastasis. Thus, OCF could be used for therapeutic applications on lung cancer in the future. Furthermore, OCF provides even more research for revealing the active compounds and their mechanisms of action.
